# Correlation Between Blood Transforming Growth Factor-Beta 1 (TGF-β1) Levels and Cardiac Function Parameters Following Doxorubicin Chemotherapy

**DOI:** 10.7759/cureus.91632

**Published:** 2025-09-04

**Authors:** Mukhlis Yazid, Muhadi LNU, Sukamtoe Koesnoe, Ikhwan Rinaldi, Kevin Winston, Dhanis Putra

**Affiliations:** 1 Cardiology, Cipto Mangunkusumo National General Hospital, Jakarta, IDN; 2 Allergy and Immunology, Cipto Mangunkusumo National General Hospital, Jakarta, IDN; 3 Hematology and Medical Oncology, Cipto Mangunkusumo National General Hospital, Jakarta, IDN; 4 Internal Medicine, Cipto Mangunkusumo National General Hospital, Jakarta, IDN; 5 Internal Medicine, Bhakti Medicare Hospital, Cicurug, IDN

**Keywords:** cardiotoxicity, chemotherapy, doxorubicin, ejection fraction, transforming growth factor-β1

## Abstract

Introduction: Doxorubicin is an anthracycline-based chemotherapeutic agent that is effective against various malignancies. However, it is associated with significant cardiotoxic side effects, which may lead to a reduction in ejection fraction (EF), tricuspid annular plane systolic excursion (TAPSE), early (E) to late (A) diastolic filling velocities (E/A), early mitral inflow velocity and mitral annular early diastolic velocity (E/E'), and myocardial dysfunction as measured by global longitudinal strain (GLS). Transforming growth factor-beta 1 (TGF-β1) is a proinflammatory cytokine that plays a key role in cardiac fibrotic remodeling and is suspected to contribute to the pathogenesis of doxorubicin-induced cardiotoxicity. Currently, human studies investigating the association between TGF-β1 levels and cardiac function deterioration due to doxorubicin remain limited. The objective of this study is to assess the correlation between serum TGF-β1 levels and EF, E/A, E/E', TAPSE, and GLS in cancer patients 3-12 months after completing full-course doxorubicin chemotherapy, as cardiotoxicity often occurs less than 12 months after chemotherapy.

Methods: This research employed a cross-sectional design. Subjects included cancer patients who completed full-course doxorubicin chemotherapy at Cipto Mangunkusumo General Hospital and Dharmais National Cancer Hospital, and who met the inclusion and exclusion criteria. Serum TGF-β1 levels were measured between the third and 12th month post-chemotherapy, concurrently with cardiac function evaluation via echocardiography to assess EF, E/A, E/E’, TAPSE, and GLS. Based on the sample size calculation, the minimum number of samples needed for this study is 55 patients. Correlation analysis was performed using Pearson or Spearman tests, depending on the data distribution. Correlation analysis was considered statistically significant if the p-value was less than 0.05.

Result: A total of 56 patients were obtained for analysis. No significant correlations were found between TGF-β1 levels and EF (r = 0.308; p = 0.779), E/A (r = -0.034; p = 0.805), E/E’ (r = 0.153; p = 0.259), TAPSE (r = 0.093; p = 0.494), or GLS (r = 0.052; p = 0.704) in analysis of all 56 patients. However, a significant correlation was observed between TGF-β1 and E/E’ (r = 0.391; p = 0.033) in patients who received a cumulative doxorubicin dose ≥600 mg/m^2^ (n = 30 patients).

Conclusion: This study found no significant correlations between TGF-β1 and EF, E/A, E/E’, TAPSE, or GLS in the overall cohort. In patients who had received a cumulative doxorubicin dose ≥600 mg/m^2^, TGF-β1 correlated significantly with E/E′. As a profibrotic cytokine, TGF-β1 contributes to myocardial fibrosis, increasing stiffness and filling pressures, which may be best captured by E/E′ rather than systolic indices. These findings suggest that higher cumulative anthracycline exposure may enhance cardiotoxicity risk and highlight TGF-β1 as a potential biomarker of anthracycline-induced cardiac fibrosis.

## Introduction

Globally, cancer is recognized as one of the leading causes of mortality. According to Global Cancer Observatory (GLOBOCAN) data in 2022, an estimated 9.7 million deaths from cancer occurred globally [1]. Chemotherapy constitutes a cornerstone of oncologic treatment and has substantially improved survival outcomes among cancer patients [2]. Despite its therapeutic efficacy and wide availability, chemotherapy is associated with a spectrum of adverse effects that may compromise the overall health status of patients, particularly regarding cardiovascular function [3,4].

Among the various chemotherapeutic agents, doxorubicin, an anthracycline derivative, has been recognized for its potent antineoplastic activity across a broad spectrum of malignancies, such as leukemia, breast cancer, and ovarian cancer [5]. However, its clinical utility is often limited by dose-dependent cardiotoxicity, which may manifest as subclinical myocardial injury, structural and functional changes in the myocardium, and in severe cases, progress to dilated cardiomyopathy and congestive heart failure [6,7]. Prevention and early detection of cardiotoxicity have become critical components of comprehensive cancer care [8].

According to a retrospective study by Badheeb et al., cardiotoxicity from chemotherapy occurred in approximately 12.5% of cancer patients [9]. A study by Cardinale et al. on doxorubicin-induced cardiotoxicity reported that the majority of cardiotoxic events occurred within the first year after chemotherapy, with an overall incidence of approximately 9% [10].

Transforming growth factor beta 1 (TGF-β1), a proinflammatory cytokine, has been implicated in myocardial fibrotic remodeling [11,12]. Preclinical studies have demonstrated that inhibition of TGF-β1 signaling pathways may attenuate pathological remodeling and preserve both systolic and diastolic function [13]. Despite promising mechanistic insights, human studies exploring the association between TGF-β1 and cardiotoxicity remain limited, thereby emphasizing the novelty and clinical relevance of the present investigation.

Given these considerations, this study aims to evaluate the correlation between serum TGF-β1 levels and cardiac function parameters such as ejection fraction (EF), global longitudinal strain (GLS), tricuspid annular plane systolic excursion (TAPSE), early (E) to late (A) diastolic filling velocities (E/A), and early mitral inflow velocity and mitral annular early diastolic velocity (E/E') in adult patients receiving doxorubicin-based chemotherapy in 3-12 months post chemotherapy. Thus, this research aims to identify the prevalence of cardiotoxicity in the study population and analyze the correlation between TGF-β1 and cardiac functions post-chemotherapy.

## Materials and methods

Study design and setting

This cross-sectional prospective study was conducted at Cipto Mangunkusumo National General Hospital and Dharmais Cancer Hospital in Jakarta, Indonesia, during the period of March 2024 to April 2025. The study population consisted of cancer patients who had completed doxorubicin-based chemotherapy within a period of 3 to 12 months prior to recruitment and who met the established inclusion and exclusion criteria. Subsequently, we used consecutive samplings to obtain subjects to minimize selection bias. Based on the sample size calculation for correlation analysis, a minimal sample size of 55 patients was needed for this study.

Baseline demographic and clinical data were collected from medical records, including patient medical record number, sex, date of birth, age, comorbidities, height, weight, type of cancer, cancer staging, and cumulative dose of doxorubicin. Hematological and biochemical parameters were also documented, including hemoglobin, total leukocyte count, differential count, platelet count, alanine aminotransferase (ALT), aspartate aminotransferase (AST), and serum creatinine. The comorbidities used in this study were hypertension, diabetes, kidney disease, and liver disease.

Inclusion and exclusion criteria

Eligible participants were selected using a predetermined sampling technique. Inclusion criteria included adult patients (≥18 years) with a confirmed cancer diagnosis who had completed a full chemotherapy regimen containing doxorubicin between 3 and 12 months prior to study enrollment. Exclusion criteria were defined to eliminate confounding factors such as pre-existing severe cardiovascular disease, previous radiotherapy to the chest region, or incomplete medical records.

Data collection and procedures

Each participant underwent a single-point assessment of TGF-β1, conducted between 3 and 12 months following the completion of their doxorubicin chemotherapy regimen. The study procedures included measurement of serum TGF-β1 levels, which was performed using the enzyme-linked immunosorbent assay (ELISA) method [14], and echocardiographic evaluation was assessed using transthoracic echocardiography to measure EF, GLS, TAPSE, E/E, and E/A’. Echocardiographic assessments were conducted and interpreted by experienced cardiologists using speckle-tracking imaging techniques in accordance with the guidelines of the American Society of Echocardiography. Echocardiography was assessed by three observers. No blinding procedure was used during the study. Echocardiographic assessments were performed using Philips ultrasound systems (Philips Healthcare, Andover, MA, USA).

Statistical analysis

Descriptive statistics were employed to summarize the characteristics of study participants. Categorical variables were presented as frequencies and percentages, while numerical variables were reported as means and standard deviations for normally distributed data, or medians and interquartile ranges for data with non-normal distribution. The normality of numerical data was assessed using the Kolmogorov-Smirnov.

To evaluate the relationship between serum TGF-β1 levels and cardiac function parameters, specifically EF and GLS, correlation analysis was conducted. If the data distribution was normal, Pearson’s correlation test was used; otherwise, Spearman’s rank correlation test was applied. The strength and direction of correlation were reported using the correlation coefficient (r) along with p-values to assess statistical significance. All statistical analyses in this study were performed using IBM SPSS Statistics for Windows, Version 26 (Released 2020; IBM Corp., Armonk, New York, United States).

## Results

Characteristics of study subjects

A total of 61 patients were initially obtained. However, five patients declined to participate in the study. Thus, a total of 56 patients were included in this study (Table 1). The mean age was 46.32 ± 10.17 years. Female participants comprised 43 (76.8%) of the total study population. The mean body mass index (BMI) was 24.35 ± 5.00 kg/m^2^, and the mean body surface area (BSA) was 1.60 ± 0.20 m^2^. The most frequently observed tumor type was breast cancer, affecting 29 patients (51.8%), followed by soft tissue sarcoma in seven patients (12.6%) and non-Hodgkin lymphoma in five patients (8.9%). Comorbid conditions were present in only eight patients (14.3%).

**Table 1:** Baseline Characteristics of Study Participants (N = 56) SD: standard deviation; IQR: interquartile range; E/A: early to late diastolic filling velocities, E/E': early mitral inflow velocity and mitral annular early diastolic velocity

Variable	Value
Age (years), mean (SD)	46.32 (10.17)
Sex, n (%)	-
Male	13 (23.2)
Female	43 (76.8)
Body mass index (BMI), mean (SD)	24.35 (5.00)
Body surface area (BSA), mean (SD)	1.60 (0.20)
Tumor type, n (%)	-
Breast cancer	29 (51.8)
Soft tissue sarcoma	7 (12.6)
Non-Hodgkin lymphoma	5 (8.9)
Lung cancer	4 (7.1)
Hodgkin lymphoma	2 (3.6)
Gastric cancer	2 (3.6)
Bone cancer with metastasis	1 (1.8)
Chronic lymphoblastic leukemia	1 (1.8)
Epidermal cancer	1 (1.8)
Others	5 (8.9)
Comorbidities, n (%)	-
Yes	8 (14.3)
No	48 (85.7)
Hemoglobin (g/dL), mean (SD)	11.18 (1.80)
Hematocrit (%), median (IQR)	34.60 (30.72-38.55)
Platelets (/µL), median (IQR)	564,000 (386,000-740,000)
Leukocytes (×10^3^/µL), mean (SD)	248.23 (115.39)
Aspartate aminotransferase (AST), median (IQR) (U/L)	27.50 (23.00-36.75)
Alanine aminotransferase (ALT), median (IQR) (U/L)	26.50 (14.50-35.75)
Urea (mg/dL), median (IQR)	18.50 (14.25-28.00)
Creatinine (mg/dL), median (IQR)	0.70 (0.60-0.90)
Cumulative doxorubicin dose (mg/m^2^), median (IQR)	609 (360-1180.2)
Ejection fraction (EF) (%), median (IQR)	68 (64-75)
E/A ratio, median (IQR)	1.25 (0.16-1.75)
E/E′ ratio, median (IQR)	8.29 (3.03-15.43)
Tricuspid annular plane systolic excursion (TAPSE) (mm), median (IQR)	21 (16-31)
Global longitudinal strain (GLS) (%), median (IQR)	-18.80 (-20.37 to -15.50)
Transforming growth factor beta-1 (TGF-β1) (pg/mL), mean (SD)	68,095.14 (17,336.32)

Laboratory evaluation revealed a mean hemoglobin level of 11.18 ± 1.8 g/dL and a median platelet count of 564,000/μL. The mean leukocyte count was 248.23 ± 115.39 ×10^3^/μL. Liver function parameters were within physiological limits, with median serum glutamic-oxaloacetic transaminase (SGOT) and serum glutamic-pyruvic transaminase (SGPT) values of 27.5 U/L and 26.5 U/L, respectively. Kidney function markers showed median urea and creatinine levels of 18.5 mg/dL and 0.70 mg/dL. The median cumulative doxorubicin dose administered was 609 mg/m^2^ (range: 360-1180.2 mg/m^2^).

Cardiac function assessment showed a median EF of 68% (range: 64-75%). Diastolic function parameters included a median E/A ratio of 1.25 (0.16-1.75) and E/E’ ratio of 8.29 (3.03-15.43). Right ventricular function (TAPSE) had a median of 21 mm (16-31 mm). The median GLS was -18.80% (range: -20.37% to -15.50%). The mean serum level of TGF-β1 was 68,095.14 ± 17,336.32 pg/mL.

Correlation of serum TGF-β1 levels with cardiac function parameters

Normality testing of cardiac function parameters and TGF-β1 was performed using the Kolmogorov-Smirnov test, which demonstrated a non-normal data distribution. Therefore, all correlation analyses were conducted using Spearman’s rank correlation.

Correlation analyses were performed to evaluate the association between serum TGF-β1 levels and echocardiographic markers of cardiac function following doxorubicin chemotherapy. No statistically significant correlations were observed between TGF-β1 and cardiac function parameters, as shown by a p-value above 0.05 (Table 2).

**Table 2:** Correlation Between TGF-β1 and Cardiac Function Parameters TGF-β1: transforming growth factor beta-1; TAPSE: tricuspid annular plane systolic excursion; E/A: early to late diastolic filling velocities, E/E': early mitral inflow velocity and mitral annular early diastolic velocity. Statistical analysis was performed using the Spearman correlation test.

Variable	Correlation coefficient (r)	p-value (p)
TGF-β1 - Ejection fraction (EF)	0.038	0.779
TGF-β1 - Global longitudinal strain (GLS)	0.052	0.704
TGF-β1 - E/A ratio	-0.034	0.805
TGF-β1 - E/E’ ratio	0.153	0.259
TGF-β1 - TAPSE	0.093	0.494

Subgroup analysis: patients with cumulative doxorubicin dose ≥600 mg/m^2^ (n = 30)

Given the dose-dependent cardiotoxicity of doxorubicin, subgroup analysis was conducted in patients receiving ≥600 mg/m^2^. A statistically significant positive correlation was identified only between TGF-β1 levels and E/E’ ratio (r = 0.391, p = 0.033), suggesting a potential association with elevated left ventricular filling pressure and diastolic dysfunction. An R value of 0.391 indicates moderate positive correlation.

No significant correlations were observed with EF, GLS, E/A, or TAPSE (Table 3). In the subgroup analysis of 30 patients, no significant differences were observed in the baseline variables compared with the overall cohort of 56 patients.

**Table 3:** Correlation Between TGF-β1 and Cardiac Function Parameters in Patients With Cumulative Doxorubicin Dose ≥600 mg/m² (n = 30) TGF-β1: transforming growth factor beta-1; E/A: early to late diastolic filling velocities, E/E': early mitral inflow velocity and mitral annular early diastolic velocity Statistical analysis was performed using the Spearman correlation test.

Variable	Correlation coefficient (r)	p-value (p)
TGF-β1 - E/A ratio	-0.029	0.878
TGF-β1 - E/E’ ratio	0.391	0.033
TGF-β1 - Tricuspid annular plane systolic excursion (TAPSE)	0.238	0.206
TGF-β1 - Ejection fraction (EF)	0.153	0.421
TGF-β1 - Global longitudinal strain (GLS)	0.040	0.834

## Discussion

This study investigated the association between serum TGF-β1 levels and cardiac function in patients treated with doxorubicin. While no significant correlations were observed between TGF-β1 and standard echocardiographic markers in the overall cohort, a significant positive correlation with the E/E′ ratio emerged in patients receiving higher cumulative doses of doxorubicin (≥600 mg/m^2^). Given that TGF-β1 promotes myocardial fibrosis, leading to increased ventricular stiffness and elevated filling pressures, its effects may be more sensitively reflected by E/E′, a marker of diastolic function. In contrast, systolic indices such as EF and GLS often remain preserved during the early stages of anthracycline-induced cardiotoxicity, providing biological plausibility for the selective correlation observed.

The lack of correlation between TGF-β1 and left ventricular systolic function markers such as EF and GLS in our study suggests that TGF-β1 may not be a sensitive biomarker for systolic impairment post-doxorubicin chemotherapy. This finding contrasts with preclinical studies that have linked TGF-β1 to myocardial fibrosis and cardiac remodeling [11,12]. It is possible that in human subjects, these pathophysiological changes are more localized, subtle, or occur later than the time points measured in this study. Nevertheless, the finding of a significant correlation between TGF-β1 and E/E’ in the high-dose subgroup adds important data. E/E’ is commonly used to estimate left ventricular filling pressures and has been associated with diastolic dysfunction [15]. The association suggests that TGF-β1 may play a role in the early development of diastolic abnormalities in patients exposed to high levels of doxorubicin.

There are several possibilities why this study did not find a correlation between TGF-β1 levels and cardiac function. Firstly, it may be related to the BMI of the study population used. As described previously, the BMI of patients in this study was 24.35 (SD 5.00) kg/m^2^. According to the World Health Organization (WHO) criteria, this value falls within the normal weight category for Asian (18.5-24.9 kg/m^2^) [16]. This is important because BMI has been associated with various prognostic parameters and risks of treatment toxicity, including cardiotoxicity from anthracycline chemotherapy such as doxorubicin [17,18]. Furthermore, chemotherapy dosing is generally based on BSA, where a higher BSA typically corresponds to a higher chemotherapy dose [19]. BSA is correlated with BMI. Thus, it can be said that a higher BMI leads to a higher administered chemotherapy dose.

Several studies have shown that Caucasian individuals tend to have consistently higher BMI values compared to Asian populations. In a study by Deurenberg et al., a comparison was made between Dutch Caucasians and Chinese individuals in Beijing [20]. The results showed that Dutch Caucasians had higher BMI values. A study by Wang et al. also confirmed that Asians generally have lower BMI than Caucasian individuals but higher body fat percentages [21]. Meanwhile, research by Wen et al. reported that the mortality risk due to being overweight is higher in Asians than in Caucasian individuals at the same BMI level [22]. Thus, the chemotherapy dose in the Caucasian population is higher than in the Asian population, which may cause a higher risk of cardiotoxicity. However, in this study, the mean BMI was 24.35 kg/m^2^, suggesting that the cardiotoxicity risk may not be as pronounced as in Caucasian populations with higher average BMI. All in all, this could influence toxicity thresholds and the performance of cardiac parameters such as EF, E/E’, TAPSE, and GLS used to monitor ventricular dysfunction.

This study has several limitations that must be acknowledged. First, its cross-sectional prospective design limits the ability to establish temporal or causal relationships. Second, the single-point measurement of both TGF-β1 and echocardiographic parameters, conducted between 3 and 12 months post-chemotherapy, may not fully capture the dynamic trajectory of anthracycline-induced cardiac changes. Third, the study population was predominantly female, under 60 years of age, and had a mean BMI within the normal range for Asian populations. This relatively homogeneous demographic profile may reduce the generalizability of findings to older patients, males, or populations with higher BMI, such as Western cohorts, who are at increased risk of anthracycline-related cardiotoxicity.

Despite these limitations, the study has several notable strengths. It recruited patients across multiple centers, thereby enhancing the representativeness of the cohort. In addition, the use of a comprehensive echocardiographic assessment, including both systolic (EF, GLS, TAPSE) and diastolic (E/A, E/E′) parameters, provided a nuanced evaluation of cardiac function. Finally, the inclusion of a biomarker linked to myocardial fibrosis (TGF-β1) novelty in this study.

Future implications

Future research should aim to validate these findings in larger and more diverse populations using longitudinal designs. Serial measurements of TGF-β1 and advanced echocardiographic techniques could help clarify temporal relationships and assess whether changes in TGF-β1 levels precede, coincide with, or follow alterations in cardiac function. In addition, exploring interactions with other biomarkers of fibrosis and inflammation may enhance risk stratification and support the development of cardioprotective strategies tailored to individual patient profiles. In future longitudinal studies, serial assessments of both TGF-β1 and cardiac imaging should be performed to better capture the temporal sequence of anthracycline-induced injury. Measurements are recommended at baseline (pre-therapy), mid-therapy, and end-of-therapy.

## Conclusions

This study demonstrated that, overall, serum TGF-β1 levels were not significantly correlated with standard echocardiographic markers of cardiac function after doxorubicin chemotherapy. A significant positive correlation was observed only in the high-dose subgroup (≥600 mg/m^2^) between TGF-β1 and the E/E′ ratio, suggesting a potential link between TGF-β1 and diastolic dysfunction rather than global cardiac impairment. These findings should be considered preliminary and not yet clinically actionable. Future studies with larger cohorts and longitudinal follow-up are needed to validate the predictive value of TGF-β1, ideally in comparison with established cardiac biomarkers such as N-terminal pro b-type natriuretic peptide (NT-proBNP) and troponin. Standardization of post-chemotherapy assessment intervals will also be important to reduce variability and to clarify whether TGF-β1 can serve as an early marker of anthracycline-induced cardiotoxicity.
